# Development of a Novel Electrochemical Sensor for Determination of Matrine in *Sophora flavescens*

**DOI:** 10.3390/molecules22040575

**Published:** 2017-04-01

**Authors:** Junping Zhang, Yanchun Wang, Wei Zheng

**Affiliations:** 1 Department of Oncology, Henan Academy institute of Traditional Chinese Medicine, Zhengzhou 450000, Henan, China; zhangjunping888@163.com; 2 Department of Traditional Chinese Medicine, Henan Province People’s Hospital, Zhengzhou 450002, Henan, China; wang88982@126.com

**Keywords:** matrine, voltammetric sensors, *Sophora flavescens*, electrochemistry, hydroxyapatite

## Abstract

A simple and sensitive electrochemical sensor fabricated with graphene nanosheets (GNs) and a hydroxyapatite (HA) nanocomposite–modified glassy carbon electrode (GCE) was developed for the determination of matrine (MT). The as-prepared electrode (GNs/HA/GCE) was verified to outperform bare a GCE and GNs-modified electrode with increased oxidation peak currents and the decreased over-potential in the redox process of MT, indicating the great enhancement of electrocatalytic activity toward the oxidation of MT by the composite of GNs and HA. Under the optimized conditions, the oxidation peak currents were related linearly with the concentration of MT, ranging from 2 μM to 3 mM, and the detection limit (S/N = 3) was 1.2 μM. In addition, the proposed electrochemical sensor can be successfully applied in the quantitative determination of MT in *Sophora flavescens* extract.

## 1. Introduction

Traditional Chinese medicine (TCM), with a long history in China, has gained popularity throughout the world with successful application in clinical treatments for many diseases due to its extensive functions [1,2,3,4]. Matrine (MT), as a main component of *Sophora flavescens* Root, is a type of quinolizidine alkaloid that possesses extensive anti-inflammatory, antiparasitic, anti-arrhythmia, antibacterial, antitumor and antianaphylactic activities [5,6,7]. The herbs containing MT can be taken orally or used extensively in the synthesis of traditional Chinese medicine as well, and they are effective in the clinical treatment of various diseases such as hepatitis, acute dysentery, acute pharyngolaryngitis, eczema, gastrointestinal hemorrhage and colitis. Considering the significant pharmacological activities and extensive clinical applications of MT, developing a simple, rapid and highly sensitive method for the determination of MT of different contents in herbs is highly demanded for its medicinal preparations.

Several methods including capillary electrophoresis (CE) [8,9,10,11], gas chromatography–mass spectrometry (GC-MS) [12], high-performance liquid chromatography (HPLC) [13,14,15,16], and high-performance liquid chromatography–mass spectrometry (HPLC-MS) [17,18,19,20] have been developed for the detection of matrine and oxymatrine. The GC technique with gas as the mobile phase is suitable for the detection of organics with a low boiling point. As for the HPLC technique, the consumption of bulk solvent and the lack of specificity and selectivity due to the employment of UV detection limits its large-scale application despite its various advantages, such as excellent analytical precision and high sample loading capacity. As far as we know, few reports on the detection of MT by electrochemical techniques have been studied [21,22,23]. For example, Miao [22] investigated the electrochemical behaviors of matrine at a l-cysteine-modified electrode. The voltammetric technique can provide important information about the pharmacological activity of MT owing to its ability to identify the redox mechanism of MT. A further improvement of the selectively and sensitivity of the electrochemical electrode is essential for accurate determination of MT in real samples.

Graphene which is composed of a single layer of hexagonally bonded carbon atoms has been widely studied recently owing to its various advantages, including a high surface area and Young’s modulus, and excellent electrical and thermal conductivity [24,25,26,27,28]. Graphene exhibited unique properties that are distinctly different from carbon nanotubes (CNTs) and fullerenes owing to its two-dimensional structure [29,30]. Graphene contributes to the promotion of charge transfer owing to its subtle electronic properties, leading to the promising potential usage of graphene as an electrode material in the application of electrochemical sensors and biosensors [31,32,33,34,35,36,37,38,39,40,41,42]. The electrodes fabricated with graphene nanosheets have been proven to be effective for the determination of certain biological and organic molecules such as enzymes [43], DNA [44], small biomolecules [45], heavy metal ions [46] and gas [47]. In addition, the synthesis of composite material that is composed of graphene sheets can be another possible route to harness the excellent properties of graphene [48]. In order to achieve the successful preparation of graphene-containing composite material, graphene sheets are required to be mass-produced firstly and then distributed into various matrices homogeneously. Furthermore, the as-synthesized graphene-containing nanocomposites can be employed in various applications such as photovoltaic devices [49], Li-ion batteries [50], fuel cells [51,52], supercapacitors [53], photocatalysis [54] and capacitive deionization [55] as well.

Recently, the development of non-silica-based inorganic hybrid nanomaterials for various applications in biology, electronics and the information manufacturing industry has been intensively studied [56,57]. As an analogous to the mineral component of bone, hydroxyapatite (Ca_5_(PO_4_)_3_(OH), HA) has received wide attention for its promising potential usage in various applications such as bone cement, protein separation, adsorbents, tooth paste additives and immunosensors owing to its excellent biocompatibility and multi-adsorbing sites as a bioceramic [58,59,60,61,62]. HA nanoparticles with a high surface area are more desirable in order to obtain a better application. However, HA crystals cannot be used in electrochemical devices due to its extremely low conductivity [63,64]. It was found that the conductivity of HA could be successfully improved by the doping of other ions or nanoparticles. For instance, the nanocomposite composed of gold and hydroxyapatite has been verified to be effective as an electrochemical immunosensor for the determination of antigens [65].

In this study, the composite of graphene nanosheets (GNs) and hydroxyapatite (HA) was successfully synthesized and then used to modify a glassy carbon electrode (GCE). The electrochemical behavior of the as-fabricated electrode for the determination of MT was investigated in detail. The proposed voltammetric sensor could be employed for the determination of MT content in *Sophora flavescens* samples with high sensitivity.

## 2. Results and Discussion

**Figure 1:**
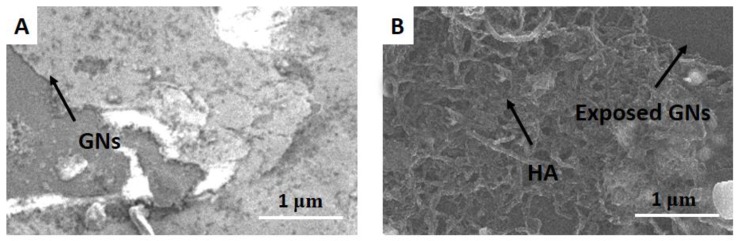
SEM images of graphene nanosheets (GNs) (**A**) and GNs/hydroxyapatite (HA) (**B**) on Si substrate.

The morphology features of GNs and GNs/HA were characterized by SEM and the results are shown in Figure 1. As shown from the SEM image of graphene sheets that were synthesized by the reduction of graphene oxide (Figure 1A), a wrinkled-paper–like morphology was observed. As for the application of graphene sheets in electrochemical sensors, the observed wrinkled nature with a high surface area is highly favourable. As can be seen from the SEM image of GNs/HA (Figure 1B), due to the sequence of the sensor fabrication process, HA was in a uniform network-structured cover on the GN surface.

**Figure 2:**
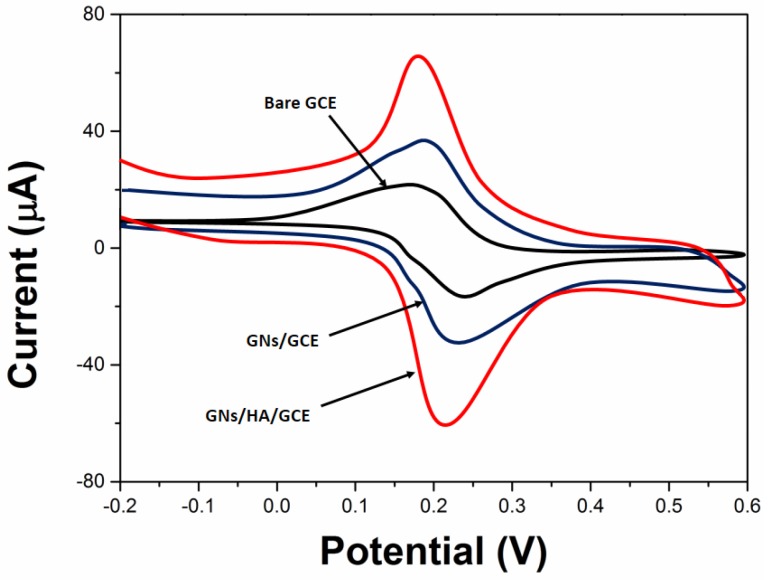
Cyclic voltammograms (CVs) of 1 mM K_3_[Fe(CN)_6_] + 0.1 M HCl on bare glassy carbon electrode (GCE), GNs/GCE and GNs/HA/GCE. Scan rate: 50 mV/s.

The charge transfer property of as-prepared GNs/HA/GCE was investigated with [Fe(CN)_6_]^3−/4−^ as a redox probe owing to its significant sensitivity to the surface chemistry of carbon-based electrodes. Cyclic voltammograms (CVs) for 1 mM K_3_[Fe(CN)_6_] that contained KCl solution (0.1 M) were performed on bare GCE, GNs/GCE and GNs/HA/GCE and the results are shown in Figure 2. The current response toward [Fe(CN)_6_]^3−/4−^ was proven to be enhanced with GNs/HA/GCE, indicating that the electrochemical active sites of GCE were greatly increased after the modification of GNs/HA. In addition, GNs/HA/GCE possessed the largest peak currents and smallest Δ*E*_p_ of the redox probe [Fe(CN)_6_]^3−/4−^, suggesting that GNs/HA outperforms GNs in the increase of the active surface area and the acceleration of the electron transfer rate.

**Figure 3:**
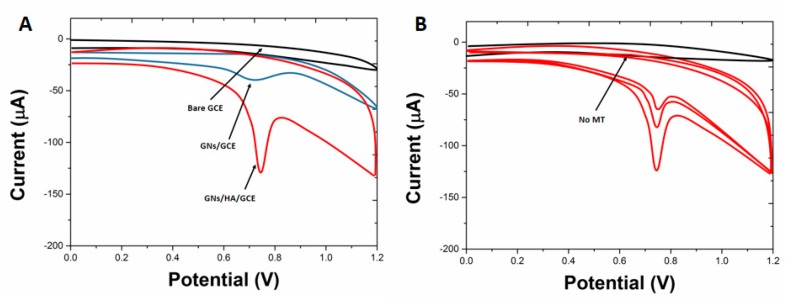
(**A**) CVs of 0.1 mM matrine (MT) obtained on bare GCE, GNs/GCE and GNs/HA/GCE, respectively; (**B**) CVs of background and 1.0 M MT at the GNs/HA/GCE. Conditions: 0.2 M PBS, pH 7.5, scan rate 50 mV/s.

The voltammetric response of the proposed GNs/HA/GCE was compared with that of other electrodes in order to investigate the effect of the GNs/HA composite. Figure 3A shows CVs of 0.1 mM MT in 0.2 M PBS solution with a pH of 7.5 obtained on bare GCE, GNs/GCE and GNs/HA/GCE, respectively. It was found that MT exhibited electrochemical activity on all electrodes. As shown from the CV obtained on bare GCE, a very small bulge was observed at 1.09 V, indicating the weak electrochemical response of MT. As to the electrochemical response of MT on GNs/GCE, the oxidation peak current (*i*_pa_) exhibited a significant increase and the peak potential (*E*_pa_) exhibited a negative shift to 0.78 V as well, suggesting that the electrochemical signal was amplified effectively owing to the acceleration of the electron transfer on the electrode surface by GNs which possess a large surface area and remarkable electric conductivity. In contrast, a distinct well-defined and more sensitive anodic peak was observed on GNs/HA/GCE among the three studied electrodes, indicating that the more efficient interface and microenvironment which are beneficial for the electrochemical response of MT could be obtained with the GNs/HA composite. It can be noticed that the oxidation potential of MT on GNs/HA/GCE is slightly higher than that of the GNs/GCE, probably due to the decrease of the electrocatalytic potential of GNs by the surface coverage of HA.

Successive CVs of MT were carried out on GNs/HA/GCE in order to investigate the redox properties of MT. CVs of the background (curve a) and 0.1 mM MT in 0.2 M PBS with a pH of 7.5 on GNs/HA/GCE are shown in Figure 3B, respectively. The anodic peak current was found to decrease obviously in the second scan compared to the first scan, and then gradually decreased with the successive cyclic sweep. The gradual decrease of the anodic peak current might result from the hindrance of the MT to the electrode surface by the formed oxidation product of MT. Moreover, the surface of GNs/HA/GCE could be restored to the initial state by the following method. Firstly, the spent GNs/HA/GCE electrode was kept in PBS with a pH of 7.5 without MT for 3 min under constant stirring, and then CV was carried out in the scan potential range from 0.0 V to 1.2 V until the disappearance of the MT peaks. The voltammogram obtained on the restored electrode was consistent with that obtained at the first cycle. Therefore, the peak current taken from the first cycle was used for the all discussions below.

**Figure 4:**
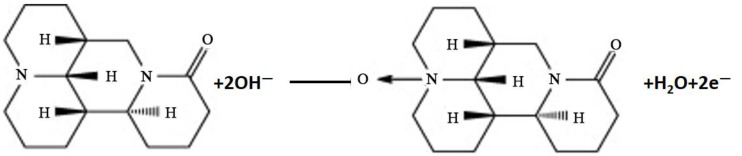
Oxidation mechanism of MT on GNs/HA/GCE.

The voltammetric response of MT was greatly influenced by the used types of supporting electrolytes. Among the studied various buffers, including citrate, acetarte, phosphate, borate and Britton-Robinson buffer, phosphate buffer (PBS) was the best with a sharp response and excellent sensitivity. Therefore, PBS solutions with the concentration of 0.1 mM and varying pH values from 5.0 to 8.5 were used in this study (Figure S1). *E*_pa_ shifted to lower values with the increasing pH. The relationship between the *E*_pa_ and pH could be fitted into the regression equation: *E*_pa_ (V) = 1.208 − 0.057 pH (*R* = 0.997). It was found that the same electrons and protons are consumed in the electrode reaction as indicated by the slope of the regression equation. Therefore, the oxidation mechanism for matrine can be written as shown in Figure 4, which is in accordance with the report [23,66]. Besides, the *i*_pa_ varies very little in the range of 7.0–7.5, so the solution pH of 7.5 was chosen in the following experiments, which is close to the human physiological pH.

**Figure 5:**
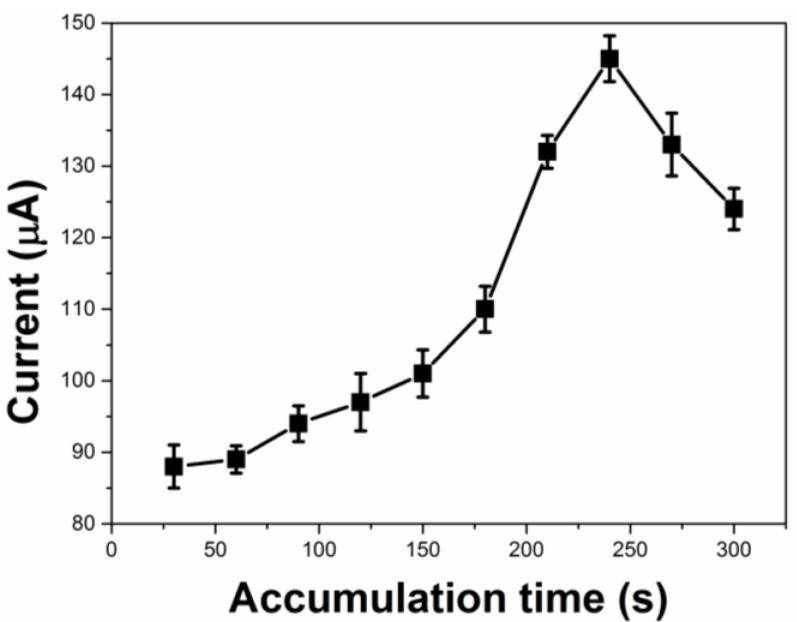
The influence of accumulation time on the current response of MT. Conditions: 0.2 M PBS, pH 7.5, scan rate 50 mV/s.

Linear sweep anodic stripping voltammetry (LSASV) was employed in the determination of MT for the sake of decreasing the background current. The scan rate of 50 mV/s was selected as the most suitable scan rate in order to enhance the sensitivity. In addition, a preconcentration could be employed to improve the detection sensitivity as to the adsorption-driven electrode reaction. The influence of the accumulation time (*t*_acc_) on the current response of 0.5 μM MT was investigated and the results are shown in Figure 5. Obviously, *i*_pa_ increased sharply within 240 s and then flattened out. A longer *t*_acc_ will lead to a better detection limit but a narrower linear range. Thus, 240 s was selected as the accumulation time by considering these factors comprehensively. Moreover, the accumulation was performed under an open circuit owing to the small effect of the accumulation potential on *i*_pa_.

**Figure 6:**
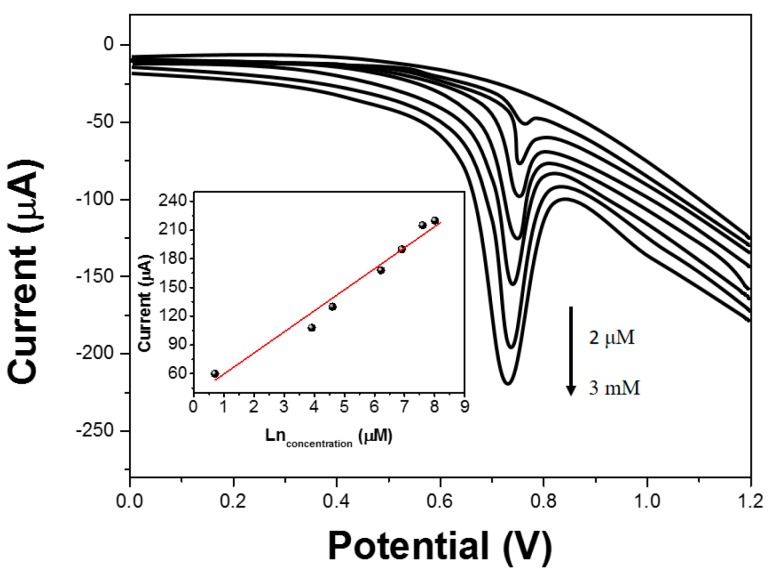
Linear sweep adsorptive stripping voltammograms and their associated calibration plot (insert) with increasing concentrations of MT at GNs/HA/GCE under optimum conditions.

MT standard solutions with a series of concentrations were determined under the optimized conditions. The response of MT at different concentrations by LSASV is shown in Figure 6. The inset of Figure 6 displays the established linear relationship between *i*_pa_ and the concentration of MT ranging from 2 μM to 3 mM. The values of the slope and intercept of the calibration curve were 0.00141 and 0.0998, respectively. The detection limit (S/N = 3) was 1.2 μM, indicating that the proposed GNs/HA/GCE was sensitive for the determination of MT. The stability, reproducibility and repeatability of GNs/HA/GCE were also estimated. The modified electrode remained at 92% of its initial current response to MT after two weeks of storage, indicating the electrochemical sensor has good stability. The reproducibility was examined at five modified electrodes prepared in the same conditions and a relative standard deviation (RSD) of 5.3% was obtained. The RSD of the response to 0.1 mM MT was 5.1% for six successive measurements, indicating the electrochemical sensor has the good repeatability. The influence of various potentially interfering substances for the determination of 0.1 Mm MT was studied. The results indicated that 20-fold of Zn^2+^, Cu^2+^ SO_4_^2−^, glucose, sucrose and amylum, ascorbic acid and uric acid had almost no influence on the determination, and the tolerance limit was estimated to be less than 7% of the relative error.

The stability, reproducibility and repeatability of GNs/HA/GCE were also evaluated. The current response of GNs/HA/GCE after 14 days of storage in the determination of MT remained at 93.7% compared with that of the initial current response, suggesting the excellent stability of the proposed electrochemical sensor. The responses of five GNs/HA/GCE electrodes prepared at exactly the same conditions to 0.1 mM MT were investigated and the relative standard deviation (RSD) was 4.3%, indicating the remarkable reproducibility of the proposed electrode. As to the same electrochemical sensor in the determination of 0.1 mM MT, the RSD of 10 successive measurements was 4.1%, suggesting the outstanding repeatability of the electrochemical sensor. In general, the excellent stability, reproducibility and repeatability of GNs/HA/GCE for the determination of MT were proven.

The proposed electrochemical sensor was employed for the determination of MT in *Sophora flavescens* extract for the sake of evaluating its validity. In order to operate in the linear range of the proposed method, the *Sophora flavescens* extract was diluted with the supporting electrolyte. After the evaluation of MT content, a standard MT solution was added into the sample and then the total MT content was determined to calculate the recovery. As shown in Table 1, the contents obtained by the proposed method were compared with that obtained by the HPLC method using the *t*-test under 95% confidence levels. No significant difference was found between the two methods, suggesting that the proposed electrochemical sensor is reliable for the quantitative determination of MT in herb samples.

## 3. Materials and Methods 

Matrine was supplied by Aladdin Chemistry Co., Ltd. (Shanghai, China) and used as received., Ca(NO_3_)_2_·4H_2_O, (NH_4_)_2_HPO_4_, graphite and ammonia were obtained from Sinopharm Chemical Reagent Co., Ltd. All other reagents were analytical reagents and used without further purification. Double distilled water was used in all experiments. The standard stock solution (2 × 10^−^^3^ M) of MT was stored at 4 °C darkly before use. Britton-Robinson (B-R) buffer solution with different pH values was employed as supporting electrolytes. Differential pulse voltammetry (DPV) and cyclic voltammetry (CV) measurements were performed at room temperature on a CHI660D electrochemical workstation (CHI Instrument Company, Shanghai) with three electrode cell containing bare or GNs/HA modified GCE (d = 3 mm), platinum (Pt) wire and Ag/AgCl as working, auxiliary and reference electrode, respectively.

The well-known modified Hummers method was employed for the preparation of graphene from purified natural graphite [67,68]. And the wet chemical method was used for the synthesis of hydroxyapatite. Typically, 150 mL of Ca(NO_3_)_2_ solution (0.5 M) was added into 20 mL of (NH_4_)_2_HPO_4_ solution (0.5 M) and the mixture was stirred at 37 °C until the clear solution was obtained. Then ammonia water was added to adjust the pH of mixture to 10. Then 70 mL of (NH_4_)_2_HPO_4_ solution (0.5 M) was added dropwise into the mixture under vigorous agitation. It was worth noting that the pH values of the mixture must be maintained at 10 by titration of diluted ammonia water during the addition of (NH_4_)_2_HPO_4_ solution. Subsequently, the mixtures were stirred for another 2 h and then aged at 37 °C for 24 h. Finally, the resulting mixture was treated with centrifugation and the obtained solid was washed and dried at 90 °C in an oven.

Then 10 mg of as-synthesized graphene was dissolved in 100 mL of deionized water and the mixture was treated with ultrasonic agitation until the homogeneous black suspension was formed. Before use, the glassy carbon electrode (GCE) with the diameter of 3 mm was firstly polished with an abrasive cloth composed of 0.3 and 0.05 μm α-Al_2_O_3_ slurry, then washed with ethanol and distilled water thoroughly under ultrasonication and finally dried with high-purity nitrogen gas. Afterwards, 5 μL of graphene solution with the concentration of 0.1 mg/mL was dropped onto the pre-treated GCE, and then the graphene-modified GCE (GNs/GCE) was obtained after being dried under an infrared heat lamp. Finally, the obtained electrode was washed with distilled water. The immobilization of hydroxyapatite was achieved by dropping 5 μL of hydroxyapatite solution (1.2 mg/mL) on GNs/GCE and the obtained GNs/HA/GCE was then dried at room temperature.

## 4. Conclusions

In conclusion, a relatively simple voltammetric sensor fabricated with GNs/HA/GCE was proposed for the determination of MT with high sensitivity and selectivity. In comparison with the previously reported method for the detection of MT, GNs/HA/GCE exhibited significant advantages of a wide linear range and a low detection limit. In addition, the proposed electrochemical sensor could be employed for the routine analysis of MT in herb samples owing to the excellent stability, reproducibility and repeatability of GNs/HA/GCE.

## Figures and Tables

**Table 1:** The contents and recoveries of matrine detected in *Sophora flavescens* (*n* = 3).

Sample	Added (μM)	Found (μM)	HPLC Result (μM)	Recovery (%)
1	10	9.97	9.99	99.7
2	50	51.02	48.17	102.04
3	100	98.45	103.93	98.45
4	200	203.66	201.05	101.83
5	300	304.51	315.66	101.50

## Supplementary Materials

The supplementary material is available online.
