# Supplementary material for: Development of a Novel Electrochemical Sensor for Determination of Matrine in Sophora flavescens
Source: Molecules. 2017 Apr 1;22(4):575. doi: 10.3390/molecules22040575 (PMC6154538; doi:10.3390/molecules22040575)

Supplementary information

Development a novel electrochemical sensor for determination of matrine in Sophora flavescens

Junping Zhanng [1], Yanchun Wang[2] and Zheng Wei [1*]

[1] Department of Oncology， Henan Academy institute of Traditional Chinese Medicine, Zhengzhou, Henan, P. R. China

[2] Department of Traditional Chinese Medicine，Henan Province People's Hospital, Zhengzhou, Henan, P. R. China

* Correspondence: weizheng_zz@126.com;

Figure S1. CVs of 0.1 mM MT obtained at GNs/HA/GCE at pH conditions from 5.0 to 8.5.

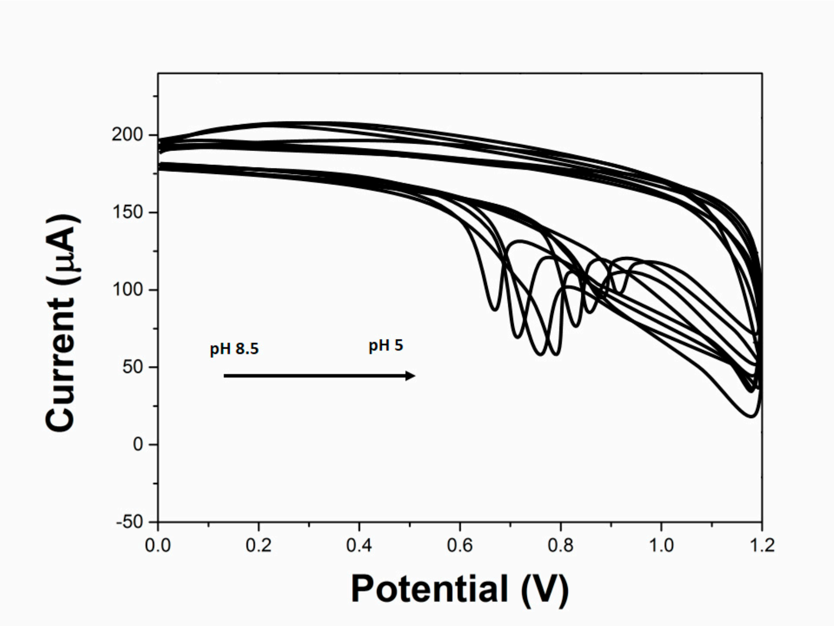

Supplement: Supplementary file 1 [file molecules-22-00575-s001.pdf]
